# Supplementary material for: Cost-effectiveness and budget impact analysis of siponimod in the treatment of secondary progressive multiple sclerosis in Italy
Source: PLoS One. 2022 Mar 8;17(3):e0264123. doi: 10.1371/journal.pone.0264123 (PMC8903273; doi:10.1371/journal.pone.0264123)
Supplement: S1 Appendix — (DOC) [file pone.0264123.s001.doc]

**S1 Appendix.** Data input details for cost-effectiveness and budget impact analysis

*Cost effectiveness analysis: data input*

***Clinical data***

The simulated cohort included in the model reflects the age, sex and EDSS level distribution reported in the EXPAND trail (Table 1 main text).

The probabilities of EDSS changes were estimated by using both data from the placebo arm in EXPAND trial and those reported the London Ontario database. To estimate the transition across different EDSS levels we used a multistate model (MSM) as previous reported for natalizumab by NICE commission.^1^

Transition probabilities between EDSS states were estimated based on the data from placebo arm of EXPAND trial.^2^ A multi-state model (MSM) approach (using the “MSM” package in R) was applied to produce the transition probability matrix, following the approach used in the natalizumab assessment conducted by NICE (TA127).^3^ A MSM was fitted using information on the EDSS level recorded at each visit scheduled during the trial, the time spent in each EDSS state and the initial values of the transition intensity matrix. When the sample size was not big enough to calculate transition probability, data from the London Ontario MS dataset was used.^4^ The transition probability matrix estimated with te MSM approach was validated comparing the model results after 1 and 2 years with the patient distribution by EDSS state observed in the EXPAND trial.

The annually probability of relapse occurrence for each EDSS level were estimated by combining: data from the placebo arm in the EXPAND trial, estimate from Patzold and Pcklington study, and data from the UK MS Survey.^2, 5, 6-7^

The mortality rate is modelled to vary overtime in line with aging of the cohort and it is estimated using all cause sex- and age-matched mortality statistics that are inflated to account for the higher mortality rate associated with MS compared to the general population. In this simulation estimation on SPMS mortality for each EDSS level was computed by using the Italian general population mortality rates as reference.^7-10^

***Treatment effectiveness***

In this cost-effectiveness study, siponimod was compared with interferon beta-1b which is indicated for the treatment of SMSP in Italy.^2, 11-13^

These treatments were associated to a significant reduction of relapse rate. While only siponimod have showed, in a randomized clinical trial, the possibility to reduce the risk of disability progression. Even if interferon beta-1b lack of clear evidence on disability progression reduction; the model assumed for both products a positive impact in reducing the diseases progression. (Table 2 main text).

The effectiveness of included DMTs was estimated performing a MAIC using data from recent literature review based on clinical trial data.^14^ The treatment efficacy was reported as Hazard Ratio (HR) using the Confirmed Disability Progression (CDP) at 6 months and as Rate Ratio (RR) using relapse rate.^15^ To estimate the reduction of relapse rate and disease progression associated to each treatment, we applied the RR and HR estimated in the review to the natural history probabilities.

We assume the probability of treatment interruption for any cause based on clinical trials reports. In the analysis data were based on EXPAND trial for siponimod and NICE guideline for interferons.

***Utility score***

The utility data used in the analysis were retrieved by a recent economic evaluation of DMTs in the multiple sclerosis patient in Italy.^16^ In that study, utility values from EQ-5D data weights for EDSS states without a relapse and during a relapse were derived from the delayed-release dimethyl fumarate clinical trial data by pooling observations for each EDSS state (0–9) and calculating the mean EuroQol EQ-5D index score for each state. The highest level of EDSS reported a negative utility value reflecting the perception of these state as worse than death for the general population.^17,18^ Negative utility value associated to EDSS 8 and 9 were frequently reported in the literature due to tremendous impact that these level of disability have on patients’ quality of life.^19,20^ In the EXPAND trials a disutility of 0.050 was applied in patients experienced a relapse for at least 49 days. The same value was adopted in our simulation independently by EDSS status.

***Resource utilization and cost***

In this study, the analysis was performed from Italian NHS perspective, therefore the cost encompassed for: overall disease management for each EDSS level; all DMTs related costs (i.e, drug cost, monitoring and follow-up costs); and cost associated to relapse.

The cost associated to disease management was retrieved from a recent Italian cost-of-illness study that used data from healthcare database and registry to estimate the economic impact of MS management. Table 1 in the main text, reported the disease costs associated with specific EDSS level. These did not include the DMTs costs that were included in the model as independent parameter. The costs were adjusted for the inflation rate reported in Italy and estimated as euros in 2019.^21^ The management costs were strictly dependent of EDSS level. Therefore, for patients with SMSP and EDSS level <7 under DMTs treatment, these included only co-medications cost, whereas for those with EDSS >7 we assumed that DMTs was discontinued therefore the cost included: co-medication costs, hospitalization, outpatients visit, and diagnostic exam not associated to DMTs.

The annual cost of DMT was estimated based on ex-factory price.^22^ The final DMTs costs were estimated by including the statutory and hidden discounts.

Administration costs associated with injectable DMTs was Euro 11.6 per administration.^23^ For interferon beta we assume that patients use the drugs under medical controls during the first three doses and then they continue with self-administration. Therefore, the costs associated with the use of interferon were those related to the aforementioned administration. The annual monitoring costs of treatment were estimated for each DMT by using the healthcare resources utilization data reported in the Emilia-Romagna Regional guidelines and in the national tariff reported by AGENAS.^16, 21, 23-25^

The costs associated to relapse of disease were estimated by using the report on healthcare resources utilization conducted by using the Administrative database of Lombardy and the multiple sclerosis center of Brescia.^21, 26^ Therefore, the cost associated to relapse’s management was 405€.

Further, costs associated to serious and non serious adverse event occurrence were estimated by considering the annual prevalence of each event. The costs associated to healthcare resources utilization were based on data reported in two NICE appraisals on MS treatment.^27^

*Budget impact analysis: data input*

***Population***

At the beginning of 2020, Italy had an estimated population of about 60 millions of inhabitants. The estimated prevalence of MS in the country is about 200 cases per 100,000 inhabitants.^28^ The aforementioned values were used to estimate the number of patients with MS in Italy, which accounted for 119.283 patients (Table 4 in the main paper).^29^ Considering the lower mortality associated to MS, the budget impact model did not account for this parameter.^21, 30, 31^

In accordance with the literature, the SPMS is about the 13.7% of all MS cases, with 91.8% of patients with SPMS age 18-60 and with an EDSS between 3-6.5.^32, 33^ Among them, 60% can be considered as affected by active form of SPMS.^32^ Finally, according with expert opinion 65.0% of patients with active SPMS can be considered as potentially eligible for DMTs.

The model included a time horizon of 3 years (study period), in which the Italian population was considered steady. However, to account for siponimod and other MS therapies diffusion, the model assumed an incremental percentage of potentially treated subjects during the study period.

***Treatments data***

In the model, siponimod was compared with the DMTs approved in Italy for the treatment of SMSP: ocrelizumab (Ocrevus ®), and Interferons beta -1b (Betaferon® and Extavia®).

***Costs associated with treatment***

The model was conducted considering the Italian NHS perspective, therefore the following costs were considered: 1) DMTs costs; 2) monitoring and follow-up costs; 3) relapse costs depending on EDSS level; and 3) AEs costs (Table A).

The annual cost of each study DMTs was reported in table 3. The cost associated with monitoring and follow-up, relapse, and AEs were the same included in the cost-effectiveness analysis.

***Market sharing***

The market share for No-Sipo scenario was estimated based on a market research and expert opinion. In this scenario, the model assumed an increased use of ocrelizumab due to recent marketing authorization in Italy and possible higher use in the next years.

Conversely, in the scenario with siponimod (Sipo), the model assumed a 6.9% of use of Siponimod during the first year followed by an increased use in the last two years, of 23.4% and 38.4% during the second and third year respectively. As reported in table B, the increased use of siponimod was balanced by reduction of other DMTs use during the same period.

**Table A.** Adverse event treatment probability and costs.

| **Treatment** | **Adverse event** | **Annual probability (%)** | **Cost (**€**)** | **Disutility** | **Source** |
| --- | --- | --- | --- | --- | --- |
| **Siponimod** | Headache | 5.08 | 1,43 | -0.0060 | 7, 10, 16, 21, 34 |
|  | Nasopharyngitis | 4.74 | 1,43 | 0 |  |
|  | Urinary tract infection | 4.21 | 36,38 | -0.0014 |  |
|  | Fall | 4.04 | 16,64 | 0 |  |
|  | Hypertension | 3.62 | 1,43 | 0 |  |
|  | Fatigue | 3.13 | 16,30 | 0 |  |
|  | Upper respiratory tract infection | 2.84 | 20,66 | 0.0041 |  |
|  | Dizziness | 2.33 | 16,64 | 0 |  |
|  | Nausea | 2.30 | 1,43 | 0 |  |
|  | Flue | 2.27 | 1,43 | -0.0002 |  |
|  | Diarrhoea | 2.17 | 0 | 0 |  |
|  | Back pain | 2.07 | 28,98 | -0.0090 |  |
|  | ALT increase | 2.01 | 1,43 | 0 |  |
|  | Peripheral Pain | 1.85 | 0,00 | -0.0058 |  |
|  | Arthralgia | 1.51 | 2,85 | -0.0078 |  |
|  | Depression | 1.51 | 419,44 | -0.0702 |  |
| **Interferon beta-1b** | Headache | 16.90 | 1,43 | -0.0060 |  |
|  | Nasopharyngitis | 9.60 | 1,43 | 0 |  |
|  | Urinary tract infection | 5.30 | 36,38 | -0.0014 |  |
|  | Infusion related reaction | 4.30 | 1,43 | 0 |  |
|  | Fatigue | 13.10 | 16,30 | 0 |  |
|  | Upper respiratory tract infection | 4.50 | 36,38 | -0.0014 |  |
|  | Back pain | 6.00 | 28,98 | -0.0090 |  |
|  | Arthralgia | 7.20 | 2,85 | -0.0078 |  |
|  | Depression | 9.00 | 419,44 | -0.0702 |  |

**Table B.** Disease-modifying therapiers (DMTs) market share in the simulated period.

|  | **Market share (%)** | | | | | |
| --- | --- | --- | --- | --- | --- | --- |
| **DMTs** | **Year 1** | | **Year 2** | | **Year 3** | |
|  | **Scenario w/o Siponimod** | **Scenario w/ Siponimod** | **Scenario w/o Siponimod** | **Scenario w/ Siponimod** | **Scenario w/o Siponimod** | **Scenario w/ Siponimod** |
| **Ocrelizuamb (Ocrevus®)** | 68.0 | 65.68 | 72.0 | 60.20 | 75.0 | 52.71 |
| **IFNB-1b 250 (Extavia®)** | 12.0 | 9.68 | 10.0 | 4.20 | 8.0 | 2.00 |
| **IFNB-1b 250 (Betaferon®)** | 20.0 | 17.68 | 18.0 | 12.20 | 17.0 | 4.62 |
| **Siponimod** | - | 6.95 | - | 23.41 | - | 38.37 |

**Figure A.** Number of subjects treated with each study drug in the observed period.

*Reference*

1. National Institute for Health and Care Excellence (NICE). Natalizumab for the treatment of adults with highly active relapsing–remitting multiple sclerosis (TA127). [www.nice.org.uk/guidance/ta127](http://www.nice.org.uk/guidance/ta127). 2007 (2007, accessed 20 March 2021)

2. Kappos L1, Bar-Or A2, Cree BAC3, Fox RJ4, Giovannoni G5, Gold R6, Vermersch P7, Arnold DL8, Arnould S9, Scherz T9, Wolf C10, Wallström E9, Dahlke F9; EXPAND Clinical Investigators. Siponimod versus placebo in secondary progressive multiple sclerosis (EXPAND): a double-blind, randomised, phase 3 study. Lancet. 2018 Mar 31;391(10127):1263-1273.

3. National Institute for Health and Care Excellence (NICE). Natalizumab for the treatment of adults with highly active relapsingremitting multiple sclerosis; 2007. https://www.nice.org.uk/guida nce/ta127/history (Accessed 20 Oct 2021).

4. Mauskopf J, Fay M, Iyer R, Sarda S, Livingston T. Cost-efectiveness of delayed-release dimethyl fumarate for the treatment of relapsing forms of multiple sclerosis in the United States. J Med Econ. 2016;19(4):432–42.

5. Patzold U, Pocklington PR. Course of multiple sclerosis. First results of a prospective study carried out of 102 MS patients from 1976-1980. Acta Neurol Scand 1982; 65: 248- 66.

6. Orme M, Kerrigan J, Tyas D, et al. The effect of disease, functional status, and relapses on the utility of people with multiple sclerosis in the UK. Value Health 2007; 10: 54-60.

7. National Institute for Health and Care Excellence (NICE). Alemtuzumab for treating relapsing‑remitting multiple sclerosis (TA312). [www.nice.org.uk/guidance/ta312/history](http://www.nice.org.uk/guidance/ta312/history). (2014, accessed 20 March 2021)

8. Pokorski RJ. Long-term survival experience of patients with multiple sclerosis. J Insur Med. 1997;29(2):101-6.

9. Harding KE, Liang K, Cossburn MD, Ingram G, Hirst CL, Pickersgill TP, et al. Long-term outcome of paediatric-onset multiple sclerosis: a population-based study. J Neurol Neurosurg Psychiatry. 2013;84(2):141-7.

10. National Institute for Health and Care Excellence (NICE). Dimethyl fumarate for treating relapsing‑remitting multiple sclerosis (TA320). www.nice.org.uk/guidance/ta320/history. (2014, accessed 20 March 2021).

11. Panitch H, Miller A, Paty D, Weinshenker B. Interferon beta-1b in secondary progressive MS: results from a 3-year controlled study. Neurology 2004; 63: 1788–95.

12. European Study Group. Placebo-controlled multicentre randomised trial of interferon beta-1b in treatment of secondary progressive multiple sclerosis. European Study Group on interferon beta-1b in secondary progressive MS. Lancet 1998; 352: 1491–97.

13. Kappos L, Weinshenker B, Pozzilli C, et al. Interferon beta-1b in secondary progressive MS: a combined analysis of the two trials. Neurology 2004; 63: 1779–87

14. Signorovitch JE1, Sikirica V, Erder MH, Xie J, Lu M, Hodgkins PS, Betts KA, Wu EQ. Matching-adjusted indirect comparisons: a new tool for timely comparative effectiveness research. Value Health. 2012 Sep-Oct;15(6):940-7.

15. National Institute for Health and Care Excellence (NICE). Ocrelizumab for treating relapsing multiple sclerosis [TA533]. www.nice.org.uk/guidance/indevelopment/gid-ta10152/documents. (2018, accessed 20 March 2021).

16. Mantovani LG, Furneri G, Bitonti R, Cortesi P, Puma E, Santoni L, Prosperini L. Cost-Effectiveness Analysis of Dimethyl Fumarate in the Treatment of Relapsing Remitting Multiple Sclerosis: An Italian Societal Perspective. Farmeconomia. Health economics and therapeutic pathways, July 2019;20(1).

17. Dolan P. Modeling valuations for EuroQol health states. Med Care. 1997 Nov;35(11):1095-108.

18. Scalone L, Cortesi PA, Ciampichini R, Belisari A, D'Angiolella LS, Cesana G, Mantovani LG. Italian population-based values of EQ-5D health states. Value Health. 2013 Jul-Aug;16(5):814-22

19. Kobelt G, Thompson A, Berg J, Gannedahl M, Eriksson J; MSCOI Study Group; European Multiple Sclerosis Platform. New insights into the burden and costs of multiple sclerosis in Europe. Mult Scler. 2017 Jul;23(8):1123-1136.

20. Kobelt G, Berg J, Lindgren P, et al. Costs and quality of life of patients with multiple sclerosis in Europe. J Neurol Neurosurg Psychiatry 2006; 77: 918–926.

21. Cortesi PA, Paolicelli D, Capobianco M, Cozzolino P, Mantovani LG. The Value and Sustainability of Ocrelizumab in Relapsing Multiple Sclerosis: A Cost-Effectiveness and Budget Impact Analysis. Farmeconomia. Health economics and therapeutic pathways 2019; 20(1): 61-72.

22. Agenzia Italiana del Farmaco (AIFA)- Italian Medicine Agency. Lists of Class A and Class H medicinal products. URL: <https://www.aifa.gov.it/liste-farmaci-a-h>. (accessed 20 March 2021).

23. Agenzia Nazionale per I Servizi Sanitari Regionali (AGENAS). www.agenas.it/prestazioni-specialistiche-ambulatoriali-confronto-tra-tariffe. (2014, accessed 20 March 2021).

24. Regione Emilia-Romagna. Assessorato Politiche per la Salute. Indicazioni per erogazione del fingolimod presso i centri sclerosi multipla dell’Emilia Romagna. www.salute.regione.emilia-romagna.it/documentazione/ptr/elaborati/159_fingolimod.pdf/view. (2012, accessed 20 March 2021).

25. Regione Emilia-Romagna. Assessorato Politiche per la Salute. Percorso regionale di diagnosi e terapia della sclerosi multipla. www.saluter.it/documentazione/ptr/elaborati/128_sclerosi_multipla.pdf. (2011, accessed 20 March 2021).

26. Cozzolino P, Cortesi PA, Cesana G, et al. The Economic Burden of Different Multiple Sclerosis Phenotypes. Value in Health 2017; 20: A721; https://doi.org/10.1016/j.jval.2017.08.1934. Poster available at: https://tools.ispor.org/RESEARCH_STUDY_DIGEST/research_index.asp. (accessed November 2019)

27. Biogen Idec Ltd, Heron Evidence Development. Natalizumab (Tysabri®) for the Treatment

of Adults with Highly Active Relapsing Remitting Multiple Sclerosis: Biogen Idec Single Technology Appraisal (STA) Submission to the National Institute for Health and Clinical Excellence. London: NICE; 2007. www .nice.org.uk/guidance /TA127/documents/multiple-sclerosis-natalizumab-manufacturer-submissions-biogen-idec-uk-andelan-pharma-international-ltd-joint-development-agreement-confidential-informationremoved2 (accessed November 2020).

28. Associazione Italiana Sclerosi Multipla – Onlus. BAROMETRO della SCLEROSI MULTIPLA 2019. https://www.aism.it/sites/default/files/Barometro_della_SM_2019estratto.pdf. (2019, accessed 20 March 2021).

29. Istituto Nazionale di Statistica (ISTAT). Demo ISTAT. Popolazione Italiana residente 1° gennaio 2019. Disponibile sul sito: http://demo.istat.it/. (accessed November 2020).

30. Atlas of MS 2013: Mapping Multiple Sclerosis Around the World. London: Multiple Sclerosis International Federation; www.msif.org/about-ms/publications- and-resources/ (2013, accessed November 2020)

31. Jick SS, Li L, Falcone GJ, et al. Mortality of patients with multiple sclerosis: a cohort study in UK primary care. J Neurol 2014; 261: 1508-17.

32. Trojano M, Bergamaschi R, Amato MP, Comi G, Ghezzi A, Lepore V, Marrosu MG, Mosconi P, Patti F, Ponzio M, Zaratin P, Battaglia MA; Italian Multiple Sclerosis Register Centers Group. The Italian multiple sclerosis register. Neurol Sci. 2019 Jan;40(1):155-165. Epub 2018 Nov 13. Erratum in: Neurol Sci. 2019 Apr;40(4):907.

33. Cortesi PA et al. The prevalence and treatment status of different Multiple Sclerosis courses in a Italian reference center. 20th Annual European Congress ISPOR 2017, Glasgow, Scotland

34. Novartis. Fingolimod for the treatment of highly active relapsing–remitting multiple sclerosis. Retrieved from NICE: <https://www.nice.org.uk/guidance/TA254/documents/manufacturer-submission-from-novartis2>. (2011, Accessed November 2020).
