# Supplementary material for: Cost-effectiveness and budget impact analysis of siponimod in the treatment of secondary progressive multiple sclerosis in Italy
Source: PLoS One. 2022 Mar 8;17(3):e0264123. doi: 10.1371/journal.pone.0264123 (PMC8903273; doi:10.1371/journal.pone.0264123)
Supplement: Attachment — Submitted filename: Reply to Reviewers comments.docx [file pone.0264123.s003.docx]

Dear Editor,

Thanks for the opportunity to address the reviewer’ comments, which was carefully considered when preparing the new revised version of the manuscript.

Our *point-by point* responses are provided below

Reviewers' comments:

**Reviewer #2:**

Thank you for addressing my comments. I'm afraid I still do not understand how you used the MAIC to adjust the disability progression matrix

We thank the reviewer for the comment. In the new version of supplemental material, we better described this aspect by including the follows sentence “The effectiveness of included DMTs was estimated performing a MAIC using data from recent literature review based on clinical trial data and EXPAND data.^14^ The treatment efficacy was reported as Hazard Ratio (HR) using the Confirmed Disability Progression (CDP) at 6 months and as Rate Ratio (RR) using relapse rate.^15^ To estimate the reduction of relapse rate and disease progression associated to each treatment, we applied the RR and HR estimated in the review to the natural history probabilities.”.

Your rationale for using CDP-6 instead of CDP-3 makes sense, although a scenario analysis using the CDP-3 results from the MAIC/European trial would help to characterize the uncertainty in your CEA.

We thank the reviewer for the comment. In the new version of manuscript, we have added a sensitivity analysis to evaluate CDP-3 as suggested by author. Therefore, we have added the following sentence in the methods “Finally, an alternative scenario analysis based on DMTs efficacy assessed using the Confirmed disability progression at 3 months (CDP-3) was performed. The CDP-3 data was retrieved by the MAIC published by Samjoo et al.12 with a Hazard Ratio of 0.74 (95%CI: 0.60-0.91) for interferon beta-1b and 0.61 (0.32-1.16) for siponimod.”, the follows one in the results “The impact of treatment efficacy was also confirmed by the alterative scenario analysis. This analysis based on CDP-3 data, siponimod reported an ICER of € 80,063 compare to interferon beta-1b.”, and finally the follows sentence in the discussion section “These results were mostly affected by DMTs efficacy, as showed by sensitivity analysis and alternative scenario analysis.”.

A few other minor comments:

-Table 1: You may want to include a citation for the dimethyl fumarate study you referenced for utilities
Amended as suggested by reviewer

-Spellcheck and review the acronyms (e.g. SMPS instead of SPMS)
Amended as suggested by reviewer
-The clarity and readability of the paper would benefit from editorial review by a native English speaker

We thank the reviewer for the comment. We revised the manuscript as suggested by reviewer. We hope the new version of paper will be clearer and more readable.
